# A vegetation classification method based on improved dual-way branch feature fusion U-net

**DOI:** 10.3389/fpls.2022.1047091

**Published:** 2022-11-29

**Authors:** Huiling Yu, Dapeng Jiang, Xiwen Peng, Yizhuo Zhang

**Affiliations:** ^1^ School of Computer Science and Artificial Intelligence, Changzhou University, Changzhou, China; ^2^ School of Mechanical and Electrical Engineering, Northeast Forestry University, Harbin, China

**Keywords:** vegetation classification, hyperspectral image, feature fusion, U-net, two-way branch network

## Abstract

Aiming at the problems of complex structure parameters and low feature extraction ability of U-Net used in vegetation classification, a deep network with improved U-Net and dual-way branch input is proposed. Firstly, The principal component analysis (PCA) is used to reduce the dimension of hyperspectral remote sensing images, and the effective bands are obtained. Secondly, the depthwise separable convolution and residual connections are combined to replace the common convolution layers of U-Net for depth feature extraction to ensure classification accuracy and reduce the complexity of network parameters. Finally, normalized difference vegetation index (NDVI), gray level co-occurrence matrix (GLCM) and edge features of hyperspectral remote sensing images are extracted respectively. The above three artificial features are fused as one input, and PCA dimension reduction features are used as another input. Based on the improved U-net, a dual-way vegetation classification model is generated. Taking the hyperspectral remote sensing image of Matiwan Village, Xiong’an, Beijing as the experimental object, the experimental results show that the precision and recall of the improved U-Net are significantly improved with the residual structure and depthwise separable convolution, reaching 97.13% and 92.36% respectively. In addition, in order to verify the effectiveness of artificial features and dual-way branch design, the accuracy of single channel and the dual-way branch are compared. The experimental results show that artificial features in single channel network interfere with the original hyperspectral data, resulting in reduction of the recognition accuracy. However, the accuracy of the dual-way branch network has been improved, reaching 98.67%. It shows that artificial features are effective complements of network features.

## 1 Introduction

### 1.1 Research background

Affected by urban development, population growth, forest fires and other factors, the protection of vegetation resources is under great pressure. Accurate identification of vegetation types and real-time control of their changes are greatly significant for environmental protection and sustainable development (Weiss et al., 2020; Ortac and Ozcan, 2021).

In recent years, the development of remote sensing technology has made it a powerful tool for vegetation resource survey and change monitoring (Wang, 2022). Before the emergence of remote sensing technology, the traditional vegetation identification methods are mostly based on field investigations, which consumes a lot of manpower and material resources. Moreover, the forest vegetation covers a large area, has a variety of vegetation types and complex terrain. These factors greatly increase the difficulty of field investigation, and cannot meet the need for updating vegetation information rapidly (Yang et al., 2022). Due to the advantages of small volume and mass, easy operation, high flexibility and short operation cycle, unmanned aerial vehicle (UAV) remote sensing system is increasingly used to obtain vegetation information quickly and accurately. At the same time, the wide application of UAV remote sensing technology also brings the progress of observation technology. Spectral images with higher resolution may lead to greater differences within the same ground objects and reduce the differences between different ground objects, that is, the confusion phenomenon of the same object with different spectrum and the different object with the same spectrum, which further increases the challenge of land cover classification of high-resolution remote sensing images. Thanks to the development of deep learning in the field of computer images, the land cover classification of remote sensing images have been gradually upgraded from the traditional manual feature design method to the automatic learning deep feature extraction method. Deep learning network extracts discriminative high-level semantic features from remote sensing images in a hierarchical manner for ground object recognition, and achieves better classification accuracy than traditional methods (Kumar and Jayagopal, 2021). Although deep learning has been widely used to study and solve the problem of high-resolution remote sensing scene classification, there are still many problems to be solved.

This paper aims to explore an accurate and fast method for extracting vegetation from hyperspectral data. Based on the design of lightweight semantic segmentation network, an improved U-Net network is designed to solve the lightweight method of semantic segmentation model without reducing the classification accuracy. By the design of multi-source spectral image fusion, the problem of low accuracy of vegetation classification of hyperspectral images is solved, which provides strong support for vegetation classification of UAV hyperspectral images.

### 1.2 Related work

In recent years, the development of remote sensing technology has made hyperspectral a powerful tool for vegetation resource investigation and change detection (Wu et al., 2017; Zhou et al., 2021). However, high-resolution spectral images will lead to greater internal differences of similar ground objects. At the same time, the differences between different ground objects will be relatively reduced, resulting in the confusion about the same object with different spectra and the different objects with the same spectrum (Kumar et al., 2022), which increases the challenge of hyperspectral image vegetation classification.

Thanks to the development of deep learning in the field of image analysis, the classification method of land cover remote sensing image, has been gradually upgraded from the traditional manual design feature method to automatic feature extraction. Deep learning extracts differentiated high-level semantic features from remote sensing images in a hierarchical manner and can obtain better accuracy than traditional classification methods (Zhong et al., 2017).

Convolutional neural network (CNN) is one of the most important directions in deep learning research. When it is used as a visual system model, it constructs a convolutional layer by imitating the characteristics of neuronal input and conductive signals in biological systems. The sample data is input to the convolutional layer for feature extraction, and the extracted feature vectors are more expressive through the activation function. Yang et al. took the high spatial resolution remote sensing imagery World View-2 of Bazhou, Hebei Province as data source, and used the deep convolution neural network SegNet to extract the rural buildings in the remote sensing image. The results show that with the Kappa coefficient of 0.90, the overall classification precision of SegNet exceeds 95%, its performance is better than the traditional classification model (Yang et al., 2019).

Lin et al. identified tree species in low-altitude aerial images based on FC-DenseNet, and the average recognition accuracy of 13 species reached more than 75% (Lin et al., 2019). U-Net is a fully convolutional network based on an encoder-decoder structure, which has concise segmentation logic and excellent segmentation efficiency (Verma et al., 2020), so it is widely used in the field of remote sensing image segmentation (Lu et al., 2021). Bragagnolo et al. classified the forest vegetation and non-vegetation areas of Amazon based on U-Net, and evaluated the forest cover change. The experimental results show that the overall classification accuracy reaches 94.7%, and U-Net can identify polygonal and fragmented forest areas (Bragagnolo et al., 2021) in a better way. Sharp U-Net (Zunair and Hamza, 2021) used deep convolution of encoder feature map with a sharpened kernel filter to generate a sharpened intermediate feature map with the same size as the encoder map to merge features of different dimensions. Compared with U-Net, which simply combines features of different dimensions by skip connection, Sharp U-Net can obtain finer grained features, thus further improving the classification accuracy.

U-Net has been widely used in the field of remote sensing image segmentation, but its ability to extract deep abstract information from hyperspectral images is limited. There are still problems in vegetation classification, such as uneven edges and misclassification (Xu et al., 2022). Deep learning methods often have the problems of large computation when dealing with high-dimensional remote sensing data. Therefore, it is of great significance to study the lightweight classification model of remote sensing images. Among them, two improved networks, Res-UNet and Mobile-UNet, are considered to be successful especially. Res-UNet introduces residual connection on the basis of U-Net, which makes the network have better feature learning ability by deepening the number of network layers. Based on U-Net, Mobile-UNet introduces depthwise separable convolution to construct lightweight deep neural network to reduce the number of parameters and operation cost. Zhu et al. proposed a land cover classification method for hyperspectral images based on a fused residual network, which used residual units to learn advanced features with more discriminative power (Zhu et al., 2021).Inspired by ResNet, Zhang et al. combined residual structure with simplified U-Net to form an RSU module (residual U-block) to extract multi-scale features and local features. The results show that the method can integrate global features while maintaining high-resolution semantic information, and improve the problem of incomplete edge segmentation of ground objects (Zhang et al., 2022).

Although remote sensing images contain rich spatial information and scale effect, which can be analyzed from different scales to obtain different levels of ground object features and spatial relationship rules, the deep learning method can only extract and recognize remote sensing images from a set scale level, lacking comprehensive consideration of multi-scale spatial information (Dalponte et al., 2018). Therefore, some researchers complement the advantages of the deep learning method and the artificial feature design method. Their effort weakens the black box feature of the deep learning method, and can obtain vegetation coverage information that is more accurate and reliable. Zhou et al. proposed artificial designed features that can provide supplementary information for CNN model in image classification tasks and put forward a framework combining CNN with Color Histogram, Histogram of Oriented Gradient, HOG, LBP Histogram, SIFT (Scale-Invariant Feature Transform), using feature encoder and joint training strategy for multi-feature fusion classification (Zhou et al., 2018). Cao et al. proposed a multi-type feature fusion classification method for hyperspectral and LiDAR. In addition to CNN features, the fusion features also include PCA, vegetation index and GLCM features of hyperspectral data, as well as DSM and intensity features of LiDAR data (Cao et al., 2018).

Taking the hyperspectral remote sensing image of Matiwan Village, Xiongan New Area as experimental object, this paper introduces residual connect and lightweight depthwise separable convolution based on U-Net framework, which replaces the traditional convolution layer of U-Net, extracts deep features, improves recognition accuracy, and reduces model complexity. In hyperspectral images, there are many types of land cover, and the boundary between vegetation classes is not obvious, which is easy to cause misclassification. Therefore, NDVI, GLCM and edge features are introduced to the deep network, and a dual-way branch input mode is designed to provide richer and more accurate feature information for the classification model, and solve the problem of insufficient features of a single type of remote sensing data. This method makes up for the deficiency of spectral information by using the spatial information and vegetation edge details provided by multi-source data, and provides support for vegetation classification method of hyperspectral images.

The main contributions are as follows:

- The residual connect and lightweight depthwise separable convolution are introduced to improve the U-Net framework for vegetable classification model, which extracts deep features, improves recognition accuracy, and reduces model complexity.
- A dual-way branch input model is designed. One branch is PCA and the other is the combination of NDVI, GLCM and edge features, which provide richer and more accurate features for the classification model.

## 2 Material and methods

### 2.1 Data source

The study area is in Matiwan Village, Xiongzhou Town, Xiongan New Area, Hebei Province, China with geographical coordinate of 38° 9 ' E, 116° 07 ' N, taken in October 2017. Data is provided by the National Data Center for Tibetan Plateau Science (http://data.tpdc.ac.cn). The terrain is higher in the northwest and slightly lower in the southeast, with an altitude of 7-19 m. It is a gently dipping plain with deep soil layer, open terrain and low vegetation coverage rate. It is located in the middle latitude zone and has a warm temperate monsoon continental climate. The research objects include 19 land cover types, among them, agricultural and forestry vegetation is the main research object. The research area has the characteristics of diverse ground objects and complex background information, which cause great challenges to the hyperspectral image classification task.

**Figure 1:**
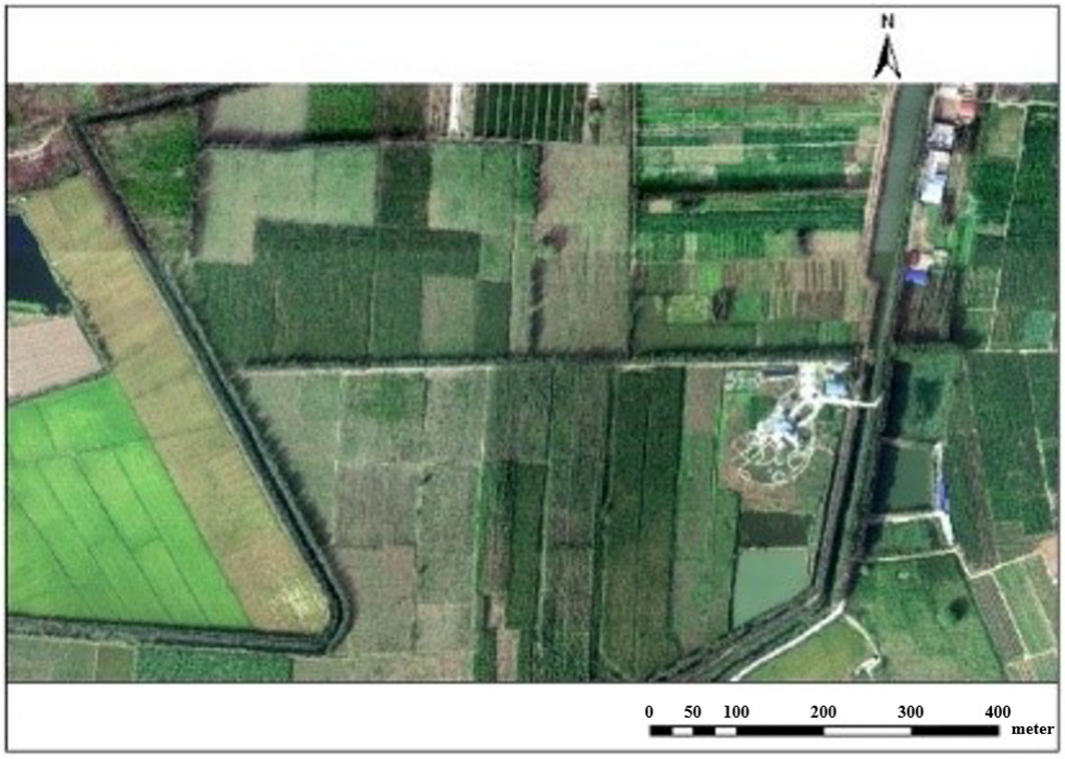
Hyperspectral image of MaTiWan Village.

The hyperspectral image is collected by the high resolution special aviation system full spectrum multimodal imaging spectrometer developed by Shanghai Institute of Technical Physics of the Chinese Academy of Sciences (Cen et al., 2020). Referring to the synchronously measured ground and atmospheric data, the pseudo color image about the reflectivity of various surface coverage types is obtained through geometric, radiometric and atmospheric correction. With a spectral range of 400-1000 nm, the image has 256 bands, and the spatial resolution is 0.5 m. The region of interest is obtained after ENVI clipping, as shown in **Figure 1**.

According to Land Use Present Situation Classification (GB/T 2010-2017), Technical Regulations for Forest Resources Planning and Design, combined with the actual land cover, we have established the land cover classification system in the study area. The classification system is used to select samples on the image. Each pixel category represents the land cover type of its location. Cover types and the number of corresponding samples are shown in **Table 1**.

**Table 1:** Vegetation classification system of Matiwan Village.

The first category	The secondary category	Number of samples
Submerged Vegetation	Rice	263453
	Rice stubble	193786
	Corn	28761
	Soybean	1303
	Vegetable field	6043
	Grassland	336000
Forest	Elm	15267
	Willow	172061
	Acer negundo	203026
	White Wax	161891
	Peach tree	60653
	Poplar	31485
	Pear tree	298658
	Robinia pseudoacacia	5612
	Goldenrain tree	20902
	Sophora Japonica	475583
Building	Building	27060
Bare area	Bare area	24953
Waters	Waters	135298

### 2.2 Dimensionality reduction based on PCA

The spectral information in hyperspectral images is rich. However, there is a certain correlation among hyperspectral bands, which may easily lead to “Hughes” in hyperspectral classification (Zhang et al., 2019). The PCA of hyperspectral images can not only improve the recognition ability of vegetation types, but also improve the computational efficiency and reduce the computational complexity.

Assuming that the number of samples of hyperspectral image is “a” and the number of bands is “b”, the hyperspectral data can be represented by matrix M. In the formula, m*_ab_* represents the value of band b in the a-*th* sample.


(1) $$M=\left(\begin{array}{ccc} m_{11} & \text{K} & m_{1b}\\ \text{M} & \text{O} & \text{M}\\ m_{a1} & \text{L} & m_{ab} \end{array}\right)$$

First, PCA gets the matrix X by standardizing M, and then calculates X covariance matrix *R*. At last, eigenvalues and the corresponding eigenvectors *R* of the covariance matrix is calculated, and the largest eigenvectors corresponding to eigenvalues are taken out, thus the desired principal components are obtained.

**Figure 2:**
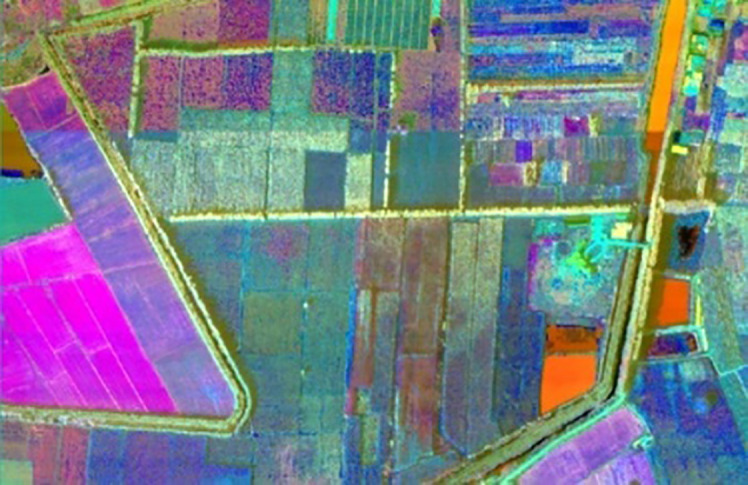
Pseudo color image after PCA.

The ENVI remote sensing analysis software is used to reduce the dimension of hyperspectral images, and PCA is performed on the original images to obtain 6-D principal component features, the hyperspectral image of Matiwan Village after dimensionality reduction is shown in **Figure 2**.

### 2.3 The improved U-Net

Depth-wise separable convolution decouples the correlation between the ordinary convolution space and dimensions. The ordinary convolution process is divided into depthwise convolution and pointwise convolution, reducing the complexity of model calculation by compressing the number of convolution kernels in convolution operation (Kulkarni et al., 2021). The residual structure enables the model to learn deeper features, enhances the propagation ability of features, extracts more ground feature details, and then improves the network segmentation ability (Zhang et al., 2019).

**Figure 3:**
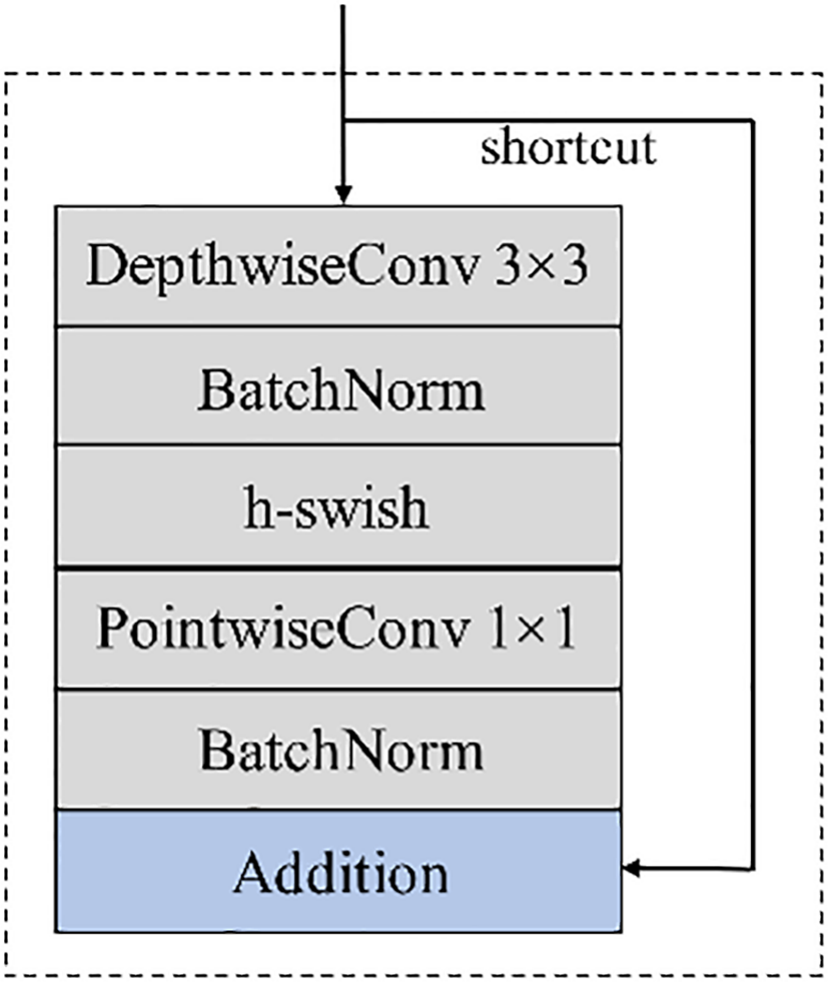
Feature extraction module.

In view of the advantages of depthwise separable convolution, we combine them to form a feature extraction module with the structure shown in **Figure 3**. After the modules are located in 3×3 depthwise convolution and 1×1 pointwise convolution, the batch normalization operation is carried out, and the input and output are directly added to learn the residual function to form the skip connection. In addition, the feature extraction module adopts the h-swish with smooth, non-monotonic and fast characteristics (Li et al., 2020; Wang et al., 2022), and the formula of h-swish is (2).


(2) $$\text{h-swish}(x)=x\frac{\text{ReLU}6(x+3)}{6}$$

In the training process, the classification of each pixel is treated as a binary classification problem with only two cases of 0 and 1 that need to be predicted by the model. For each category, the predicted probability is ${\hat{y}}_{l}$ and $1-{\hat{y}}_{l}$ , and the binary cross-entropy loss function is as follows:


(3) $$\text{BCELoss=}-\frac{1}{m}\sum_{i=1}^{m}\left[y_{i}\log\left({\hat{y}}_{l}\right)+(1-y_{i})\log\left(1-{\hat{y}}_{l}\right)\right]$$

In formula(3), *m* is the sample size, *y*_*i*_ is the label of sample *i*, and ${\hat{y}}_{l}$ is the predicted value of sample *i*.

**Figure 4:**
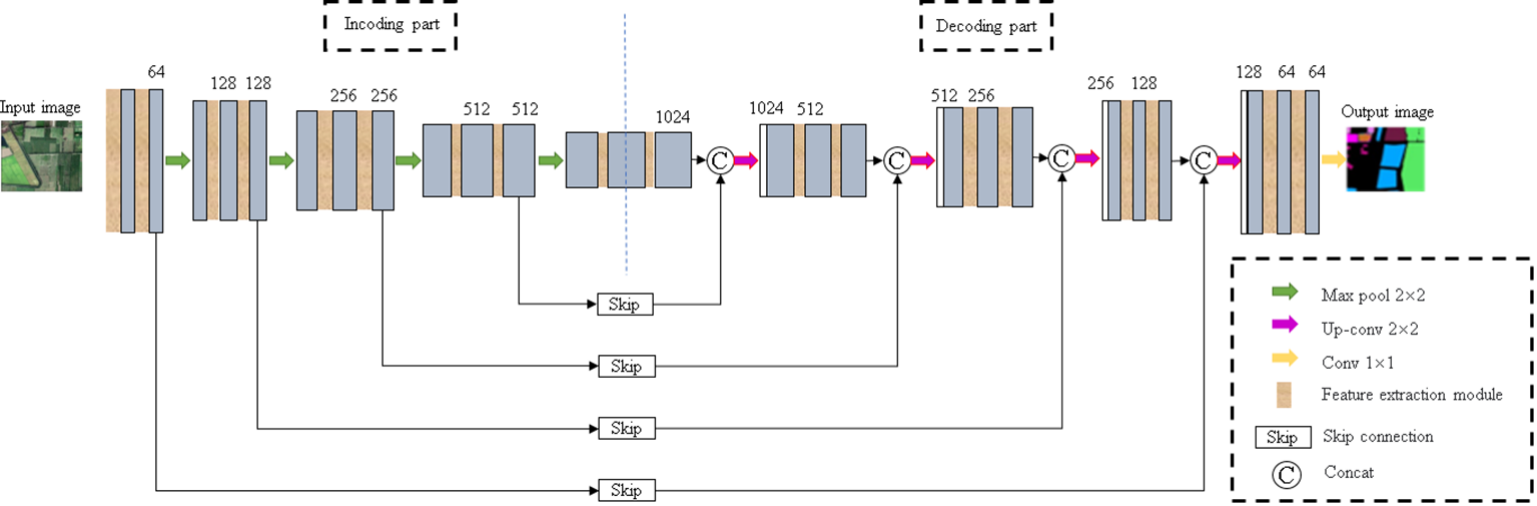
Improved U-Net model.

The improved U-Net model is shown in **Figure 4** The network consists of encoding part, decoding part and skip connection. Among them, the encoding part and the decoding part both contain five layers, and two feature extraction modules are added to each layer. The symmetric decoding and encoding part form a U-shaped structure. In the encoding part, features are extracted through the feature extraction module, and 2×2 max pooling is repeatedly used for down-sampling to extract image features from the context. In the decoding part, the proposed module is also used to replace the convolution layer in the U-Net. In order to ensure the same resolution in the fusion, 2×2 up-sampling is performed on the basic feature map in front of each layer to restore the image size. In the last layer, each pixel is classified by 1×1 convolution. In the skip connection, the features extracted from the encoding and decoding parts are fused to ensure a better combination of shallow detail information and deep background semantic information.

### 2.4 Artificial features extraction

#### 2.4.1 Normalize difference vegetation index (NDVI)

NDVI can partially remove or weaken the impact of satellite observation angle, solar altitude angle, topographic relief, and the impact of small amount of cloud shadow and atmospheric radiation on image (Garcia-Salgado and Ponomaryov, 2016). It is a surface vegetation measurement index widely used in vegetation and plant phenology research. This index is in direct proportion to the coverage of surface vegetation, and usually detects the vegetation growth status and vegetation coverage. Because the low vegetation and trees in the hyperspectral data of the research object account for a large proportion of pixels, and the distribution is irregular and interspersed around buildings and waters, NDVI can be used to reflect the vegetation coverage, so as to distinguish vegetation and non-vegetation features.

The formula of NDVI is:


(4) $$\text{NDVI}=\frac{\text{NIR}-R}{\text{NIR}+R}$$

In formula(4), NIR is near infrared band, *R* is Gray value of red band.

**Figure 5:**
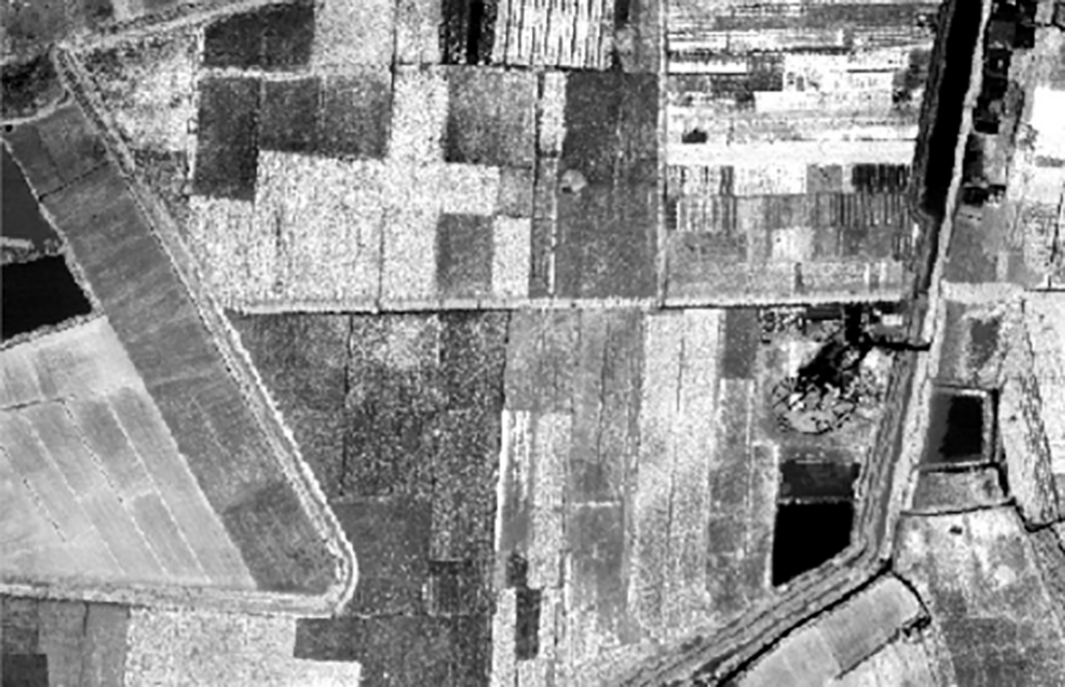
NDVI of the study area.

The range of NDVI is [-1,1]. When NDVI is positive, it indicates that there is vegetation coverage, which increases as the coverage expands. When NDVI is negative, it indicates that the ground is covered by clouds, water, snow, etc., which is highly reflective of visible light. When NDVI is 0, it indicates rock or bare soil, etc., at the same time, NIR and *R* are approximately equal. The NDVI calculated by ENVI5.3 is shown in **Figure 5**.

#### 2.4.2 Gray level co-occurrence matrix (GLCM)

**Figure 6:**
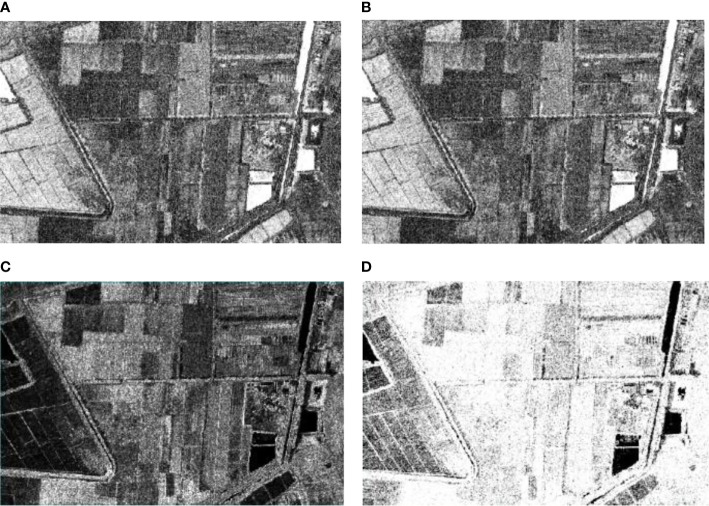
GLCM for Homogeneity, Mean, Dissimilarity and Entropy: **(A)** Homogeneity; **(B)** Mean; **(C)** Dissimilarity; **(D)** Entropy.

Texture reflects the gray distribution of pixels in the image and their surrounding spatial neighborhood. The surface characteristics of image scenery can be well described by using texture features (Mei et al., 2016). GLCM is a widely used texture analysis method. The parameters such as similarity, mean, homogeneity and entropy with clear results are selected as the texture features of the classification model. For PCA transformed images, the window size is set to 3 × 3. Based on the window size above, the parameters such as dissimilarity, mean, homogeneity and entropy are calculated to obtain the texture feature image of hyperspectral data. As shown in **Figure 6**.

#### 2.4.3 Edge features

**Figure 7:**
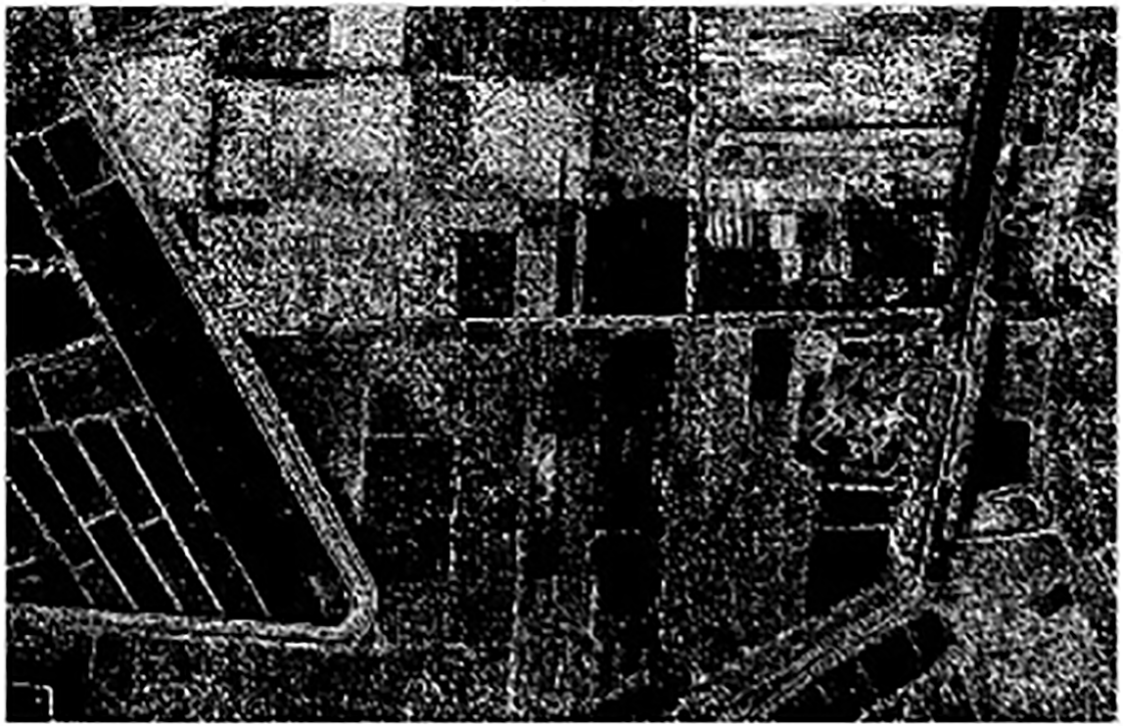
Edge detecting based on Sobel.

In image processing, the edge of the image is the region where the most obvious gray value changes could be seen. Image edge detection can reduce the amount of data significantly and retain important structural attributes in the image (Zhao and Du, 2016). Here we use Sobel to detect the image edge. The transverse and longitudinal Sobel convolution factors are shown in formula(5) and formula(6), respectively, and the experimental results are shown in **Figure 7**:


(5) $${\text{sobel}}_{x}=\left[\begin{array}{ccc} -1 & 0 & 1\\ -2 & 0 & 2\\ -1 & 0 & 1 \end{array}\right]$$

(6) $${\text{sobel}}_{y}=\left[\begin{array}{ccc} -1 & 2 & -1\\ 0 & 0 & 0\\ 1 & 2 & 1 \end{array}\right]$$

### 2.5 The improved U-Net with dual-way branch

**Figure 8:**
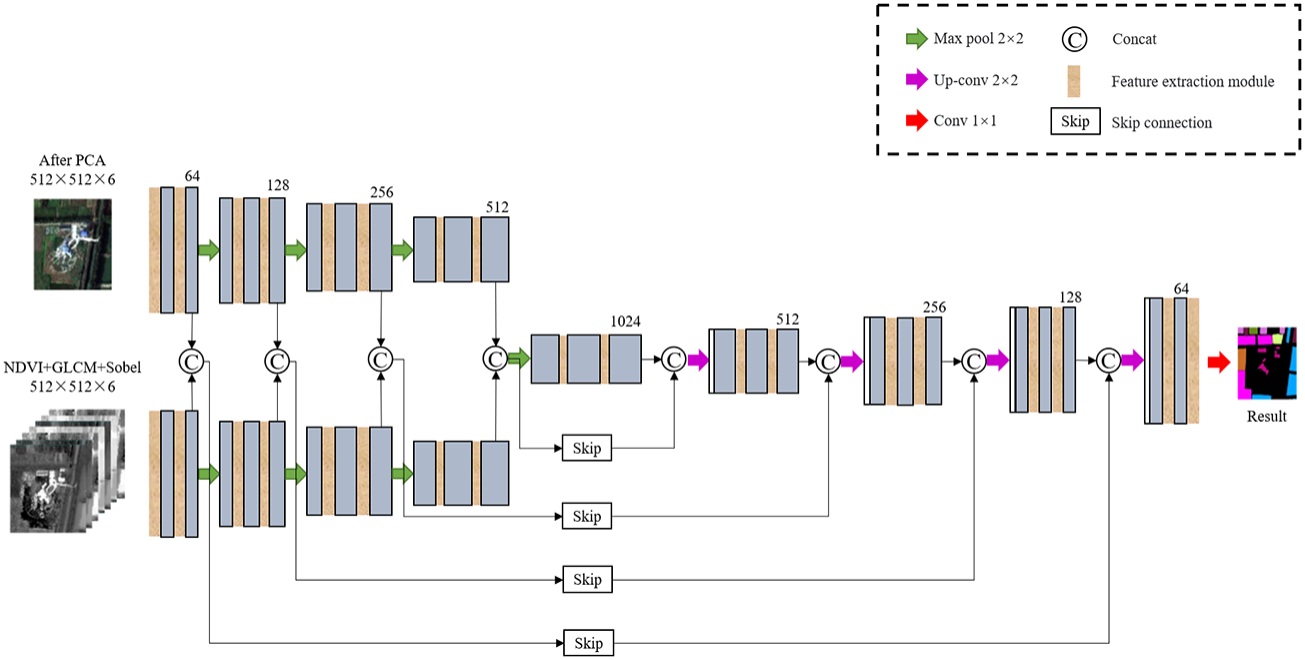
The dual-way input model based on improved U-Net.

The structure of improved network is shown in **Figure 8**. The network input is composed of two input terminals. The upper end is the hyperspectral image data after PCA dimensionality reduction, and the other end is the manually extracted NDVI, GLCM and the data image of edge features obtained by Sobel through concat operation, all sized by 512 × 512 × 6. The backbone network is U-Net, which has 4 times up-sampling and 4 times down-sampling. For the multi-source data input, the model uses a feature extraction module combining residual structure and depthwise separable convolution in the down-sampling process. After each down-sampling, the concat operation is used for feature fusion first. Then the spectral spatial semantic features and texture detail semantic features, which are extracted from multi-source data by hierarchical fusion of shared decoder, are used to improve the inter class difference and intra class consistency, and help the model to maintain the fine granularity between the edges of vegetation categories during the scale restoration of feature map. In the up-sampling phase, restore the feature map through 2×2 up-sampling, then carry on concat feature fusion of shallow features and deep features by skip connection. Among them, deep features of the up-sampling part are extracted by the feature extraction module. Finally, the soft classifier is used to judge the category of pixels.

## 3 Experiments and results analysis

The computer used in the experiment is configured as NVIDIA Quadro RTX 5000, Intel(R) Core™ I9-1085h. Under Windows operating system, based on PyCharm2019.2.3, using python3.7, run the experiment through pytorch framework. In the experiment, the hyperspectral image after PCA dimensionality reduction is divided into 512 × 512, and divided into training set and test set according to the ratio of 8:2. Set the sample set of batch training to 4, the maximum number of training iterations to 600, and the initial learning rate of the network to 0.0001. When the epoch is equal to 100, the learning rate becomes 0.0001, which makes the network find the local optimal solution; The initial weight is the pre-training weight of ImageNet.

### 3.1 Analysis of network optimization performance

**Figure 9:**
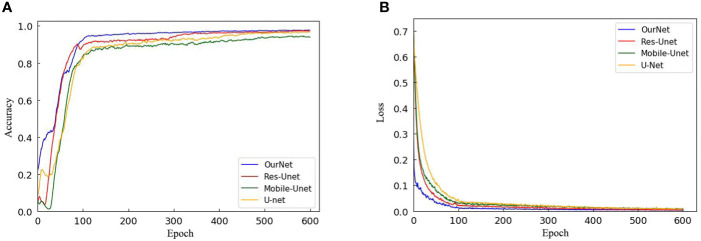
Comparative experiment of training process: **(A)** Conversion curve of each model during training; **(B)** Loss function transformation curve of each model.

To verify the segmentation performance of the improved model, the classification accuracy of U-Net, Res-UNet, Mobile-UNet and the improved U-Net are compared with hyperspectral images after PCA dimensionality reduction. **Figure 8** shows the curve of the accuracy and loss function with the number of iterations during the training process. **Table 2** shows the accuracy of the test set segmentation results, parameters, train time and test time of each network model. It is seen from **Figure 9A** that the highest accuracy is obtained from the model training method (Our-Net) proposed in this paper. Res-UNet is similar to its accuracy, followed by U-Net, and accuracy of Mobile-UNet is the lowest. In addition, compared with the other three models, the improved U-Net tends to be stable after about 100 training iterations, and then get to convergence within the shortest time. According to **Figure 9B**, the fastest loss reduction is obtained from the improved model.

**Table 2:** Comparison of precision, recall, parameters, train time and test time of four different models.

Model	Precision(%)	Recall(%)	Parameters/M	Train time/s	Test time/s
our-Net	0.9713	0.9236	6.633	17 825	17.2
Mobile-UNet	0.9266	0.8984	6.633	17 413	16.9
Res-UNet	0.9668	0.8767	24.45	23 309	20.8
U-Net	0.9421	0.8372	13.40	19 861	19.3

**Table 2** shows that depthwise separable convolution can improve the computational efficiency of the model significantly, but it reduces the classification accuracy of the network at the same time; The residual structure requires the model to learn deeper features, and then improves the network segmentation ability, making up for the lack of feature extraction ability of depthwise separable convolution. The shortcut connection of residual unit does not introduce additional parameters during network training, and will not add additional calculations to the network.

### 3.2 Improved U-net with features fusion in single channel

**Figure 10:**
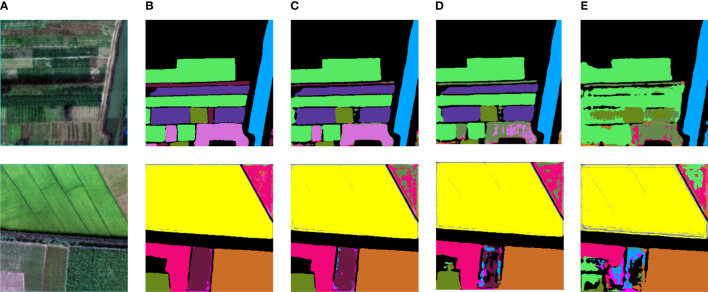
Training results of different multi-source data superposition on the improved U-NET: **(A)** Original spectral image; **(B)** PCA; **(C)** PCA+NDVI; **(D)** PCA+NDVI+Sobel; **(E)** PCA+NDVI+Sobel+GLCM.

**Figure 11:**
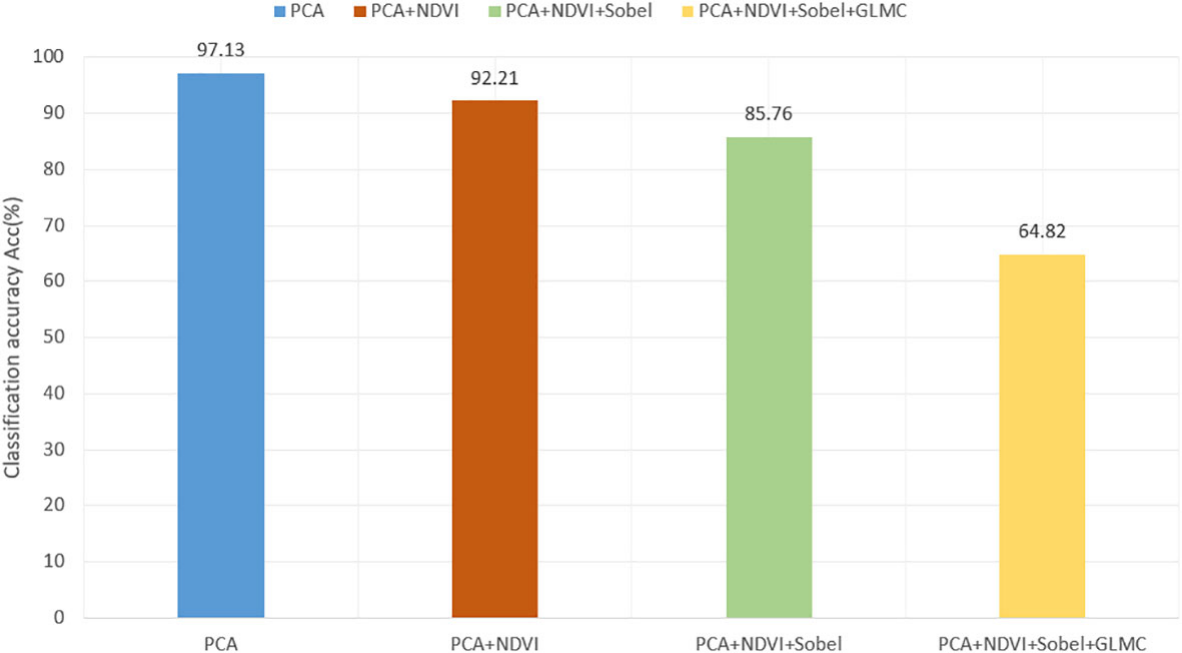
The comparison of overall classification accuracy of different feature fusion ways in single channel.

The hyperspectral images after PCA and different artificial features are obtained respectively, and then trained in the form of single branch input to the improved U-Net. **Figure 10** shows the visual prediction results of some test sets. **Figure 11** shows the overall classification accuracy of different feature fusion ways in single channel.

It can be seen from **Figure 10** that the effect of vegetation classification is the best when the original dimension reduced hyperspectral image is used in the single branch network. As the number of input channels increases, the classification effect decreases. The accuracy of vegetation classification by overlaying and fusing NDVI+Sobel+GLCM is 20.94% and 27.39% lower than the experimental results by fusing NDVI and Sobel+NDVI respectively, and 32.33% lower than the prediction results by using the original hyper-spectral data as input. In **Figure 10E**, it can be seen that some vegetation could not be recognized. The major reason for the decline of segmentation accuracy is that hyperspectral images have rich spectral information, and there will be some interference between the original hyperspectral data and artificial features, which will affect the accuracy of classification models. Therefore, multi-source data cannot be simply superimposed directly on a single source network.

### 3.3 Improved U-net with dual-way input

In **Table 3**, No.1 is the input image of the single branch network, which is the hyperspectral image of the original Matiwan Village after PCA dimensionality reduction. No.2 means that in a dual branch network, one input data source is PCA and the other is NDVI. The input of No.3 and 4 is similar to No.2, among them, one input in the network is PCA, and the other is NDVI+GLCM and NDVI+GLCM+Sobel.

**Table 3:** The influence of different feature fusion ways on experimental results.

No.	feature fusion	F1 Score(%)					Acc(%)
		Buildings	Forest	Low Vegetation	Waters	Bare areas	
1	PCA	98.05	96.32	96.64	98.83	96.52	97.13
2	PCA/NDVI	97.92	97.63	97.52	98.86	96.55	97.47
3	PCA/NDVI+GLCM	98.57	98.25	98.01	99.14	98.68	98.33
4	PCA/NDVI+GLCM+Sobel	98.59	98.58	98.20	99.25	98.73	98.67

By comparing the prediction results of No. 2 with those of No.1, it can be seen that the prediction results of Experiment 2 are 0.88% and 1.31% higher than those of No.1, except that the F1 scores of low vegetation and trees, the scores of other features are almost unchanged, the overall classification accuracy is improved by 0.34%. This is because NDVI data can only distinguish vegetation from non-vegetation, but it is difficult to make further distinctions.

In terms of classification accuracy, the F1 score and Acc of No. 3 are improved, and the overall accuracy is 1.2% and 0.86% higher than that of No.1 and No. 2 respectively. This is because adding texture features can express the spatial scale and spatial structure information of images in a better way. For objects such as waters and bare areas with obvious differences in texture features, the classification accuracy of texture data is greatly improved than that of original data. However, the F1 score of buildings doesn’t improve significantly, the main reason is the small number of samples of buildings in the selected hyperspectral data and the uneven distribution of the number of pixels in each coverage category.

Comparing the results of No.4 and No.3, it can be seen that the vegetation classification results with edge features have improved in F1 score and Acc. F1 score of building, forest, low vegetation, waters and bare areas increased by 0.02%, 0.33%, 0.19%, 0.11% and 0.05% respectively. The addition of edge features makes the network model perform better in distinguishing the details of vegetation edges.

To sum up, it can be seen that the addition of GLCM has a significant impact on the classification results of the model. It helps the model to distinguish the ground objects that are difficult to distinguish in terms of spectral and spatial characteristics, and makes the network model more accurate in distinguishing waters, buildings and bare areas. The dual-way branch combination of PCA and NDVI, GLCM, and Sobel not only provides spatial feature information, but also makes contributions to feature extraction in land class boundary recognition, shape attribute and physical quantity description, which makes the classification results more accurate, and makes up for the loss of semantic feature edge detail information.

**Table 4** shows the statistics of classification accuracy using PCA+NDVI+GLCM+Sobel multi-source data. The classification accuracy of several types of ground objects with small sample size is not high, such as soybean, vegetable field and robinia pseudoacacia. In addition, as the spectral similarity between elm, sophora japonica, maize and acer negundo is high, it shows the phenomenon of hyperspectral “different body with same spectrum”, so, there is misclassification in it, which has a certain impact on the classification accuracy.

**Table 4:** Overall classification accuracy assessment of different categories based on improved U-Net model.

Category	Acc(%)	Category	Acc(%)
Rice	99.62	Populas	96.25
Rice stubble	98.76	Pear	99.71
Corn	88.63	Robinia pseudoacacia	78.57
Soybean	73.34	Goldenrain tree	96.81
Vegetable field	81.22	Sophora Japonica	95.38
Elm	87.01	Building	98.63
Willow	98.97	Lawn	98.96
Acer negundo	95.75	Bare areas	98.73
White Wax	99.26	Waters	99.25
Peach	98.42		

**Figure 12:**
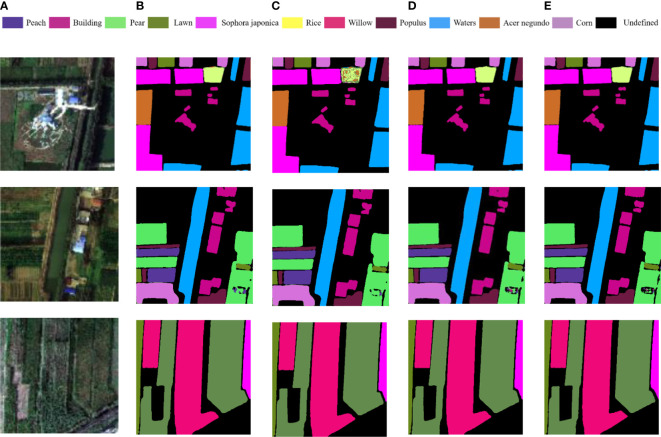
Comparison of experimental results of different data features superposition methods: **(A)** Test set; **(B)** PCA; **(C)** PCA+NDVI; **(D)** PCA+NDVI+GLCM; **(E)** PCA+NDVI+GLCM+Sobels.

Select 3 images randomly in the test set for display, as shown in **Figure 12**. It can be seen that the dual-way branch with the multi-features fusion method proposed in this paper has the best vegetation classification effect. It can not only distinguish the vegetation types more accurately, but also describe the edges and details of different vegetation areas in a better way, and the segmentation result of the coverage boundary is more obvious.

## 4 Conclusions

In this paper, hyperspectral images are used to obtain representative feature parameters, such as spatial information, texture information, edge information, etc. And the classical semantic segmentation model, U-Net, is improved. The features automatically extracted by the deep learning model and artificial features are fused for vegetation classification. The main works are as follows:

The dimension of hyperspectral image is reduced through PCA, and the band combination of effective image containing the most spectral information is obtained. The NDVI and GLCM of the image are calculated to obtain the spatial spectral features and texture features of hyperspectral image, and the edge features are calculated by Sobel; A feature extraction module is proposed, which uses depthwise separable convolution instead of traditional convolution in U-Net to extract multi-scale features of hyperspectral images, reduces network complexity, and introduces residual connection to extract deep semantic information to improve classification accuracy. Finally, a dual branch multi-source data feature fusion method is proposed for vegetation classification. The experimental results show that the method studied in this paper has advantages in overall accuracy. The dual-way branch data fusion effectively avoids the mutual interference between different data types. The advantages of hyperspectral and artificial features have been brought into full play. The addition of different artificial features can improve the accuracy in the classification of different covers, and the model can identify the boundary of vegetation in a more accurate and clear way. This vegetation classification method is practical.

In addition, due to a large number of hyperspectral feature types and uneven distribution of samples in each coverage category, how to preprocess the data set to improve the difference between spectra, and how to amplify the data of small sample categories to improve the overall classification accuracy will be the focus of future research.

## Data availability statement

The original contributions presented in the study are included in the article/supplementary material. Further inquiries can be directed to the corresponding author.

## Author contributions

YZ conceived of the presented idea and took the lead in wiring the paper. HY, DJ, and XP developed the theory and performed the computations. HY and XP wrote the paper. All authors contributed to the article and approved the submitted version.

## Conflict of interest

The authors declare that the research was conducted in the absence of any commercial or financial relationships that could be construed as a potential conflict of interest.

## Publisher’s note

All claims expressed in this article are solely those of the authors and do not necessarily represent those of their affiliated organizations, or those of the publisher, the editors and the reviewers. Any product that may be evaluated in this article, or claim that may be made by its manufacturer, is not guaranteed or endorsed by the publisher.
